# Interaction between age and atrial fibrillation on ischemic stroke severity: a cross-sectional analysis

**DOI:** 10.3389/fstro.2026.1706746

**Published:** 2026-02-02

**Authors:** Daniel Zhihao Hong, Rehena Sultana, Nur Sarah Binte Ibrahim, Hui Meng Chang, Deidre Anne De Silva

**Affiliations:** 1 Ministry of Health Holdings, Singapore, Singapore; 2 Duke-NUS Medical School, Singapore, Singapore; 3 National Neuroscience Institute, Singapore General Hospital Campus, Singapore, Singapore

**Keywords:** age, atrial fibrillation, ischemic stroke, NIHSS (National Institute of Health Stroke Scale), stroke severity

## Abstract

**Background:**

While atrial fibrillation (AF) and older age are established independent risk factors for greater ischemic stroke severity, their interactive effect remains poorly characterized. This study aimed to evaluate the interaction between age and AF status on stroke severity, as measured by the National Institutes of Health Stroke Scale (NIHSS).

**Methods:**

We conducted a cross-sectional study using a prospectively collected institutional stroke registry comprising 5,044 patients with acute ischemic stroke from 2019–2023. The primary exposures were AF status and age, categorized as < 65 and ≥65 years, while stroke severity at admission measured by the NIHSS served as the outcome. NIHSS was modeled using a negative binomial distribution within PROC GLIMMIX with a logarithmic link function to account for overdispersion in the NIHSS scores. Model included age, AF, and their interaction term, along with relevant co-variates such as gender, race and premorbid mRS.

**Results:**

AF was present in 17.3% of patients. Stroke severity was significantly greater in patients with AF across both age groups: IRR 1.88 (95% CI, 1.44–2.45; *p* < 0.0001) in patients < 65 years, and IRR 2.13 (95% CI, 1.89–2.39; *p* < 0.0001) in patients ≥65 years. The interaction between AF and age on stroke severity was not statistically significant (*p* for interaction = 0.40).

**Conclusions:**

AF is associated with greater stroke severity in both younger and older adults. There was no statistically significant interaction between age and AF status on NIHSS scores, indicating no evidence of effect modification by age within limits of our data. These findings underscore the need to consider stroke severity in risk stratification, especially in younger AF patients who often experience greater treatment burden.

## Introduction

Ischemic stroke remains one of the leading causes of disability worldwide (Feigin et al., 2025, 2021). While both old age and atrial fibrillation (AF) are well-established independent risk factors associated with worse stroke outcomes, their interactive effect, particularly in younger patients, remains poorly characterized in the existing literature (Corso et al., 2014; Lang et al., 2017; Vinding et al., 2022). In the past decade, the rising prevalence of stroke in those aged ≤ 65 has made stroke in younger populations an emerging clinical concern (Pandian et al., 2023; Satapathy et al., 2025; Zhang et al., 2025). According to current guidelines, younger patients with AF aged ≤ 65 years without additional stroke risk factors are not routinely recommended for anticoagulation (Hindricks et al., 2020). However, such recommendations may underestimate the true clinical burden and neurological consequences of stroke in this age group. This study aimed to evaluate the interaction between age and AF on stroke severity, as measured by the National Institutes of Health Stroke Scale (NIHSS). We hypothesized that AF contributes to increased stroke severity regardless of age, but the magnitude of this effect may differ by age.

## Methods

### Study design and population

**Figure 1:**
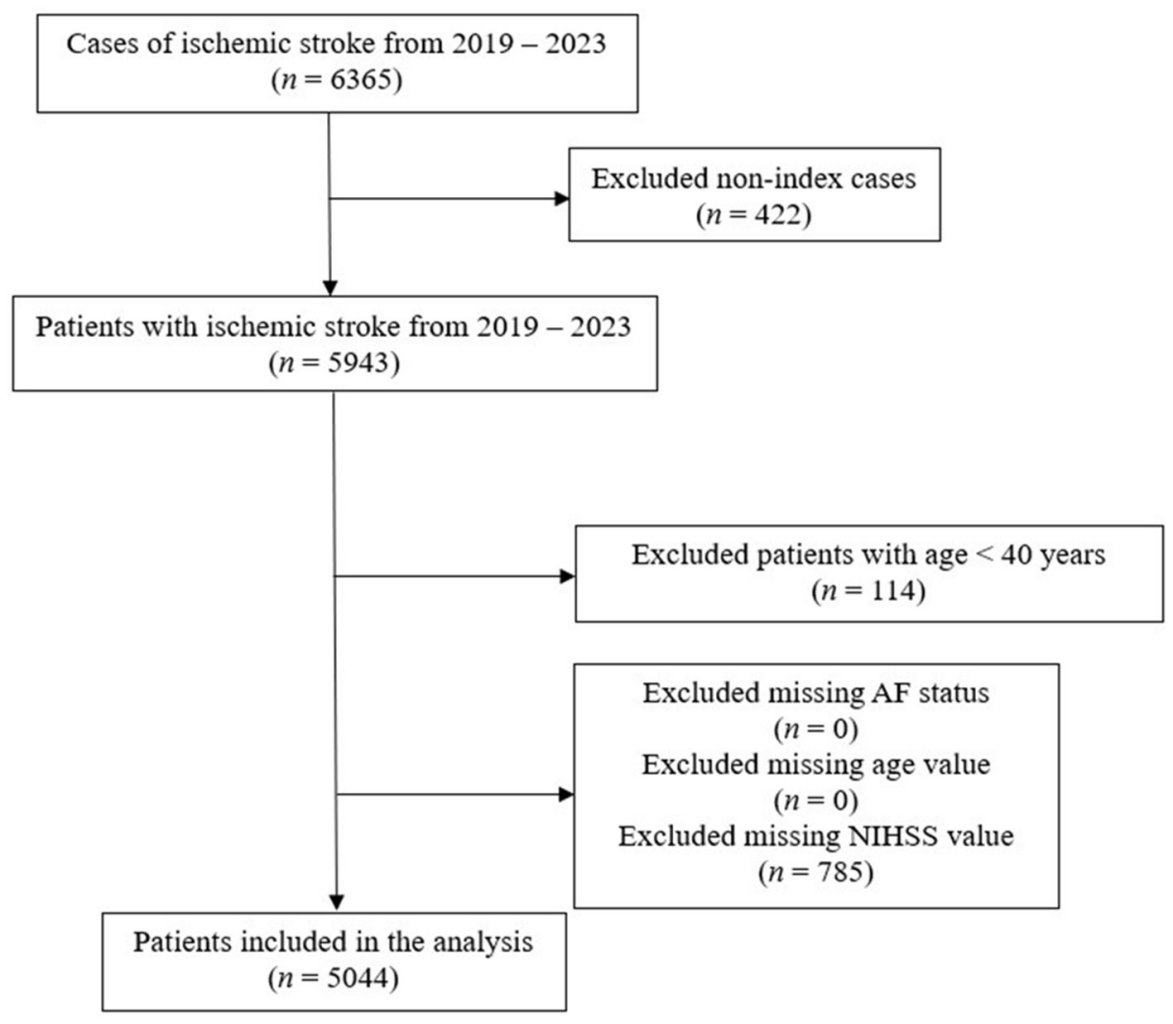
Selection of study population.

This was a cross-sectional study using data from a prospectively collected institutional stroke registry in Singapore between January 1, 2019, and December 31, 2023 (Figure 1). The dataset comprised patients admitted to a tertiary academic stroke center with a confirmed diagnosis of acute ischemic stroke. The inclusion criteria were ischemic stroke defined using International Classification of Diseases, 10th Revision, Australian Modification (ICD-10-AM) codes for cerebral infarction (I63.0–I63.9) or stroke not otherwise specified (I64) that were assigned after hospital discharge by trained coders using a standardized coding guideline. Patients were included if they had any of the aforementioned ischemic stroke codes recorded as a primary diagnosis in any inpatient location (excluding rehabilitation medicine) or as a secondary diagnosis provided that they were admitted to the acute stroke unit under neurology care. To avoid duplicate inclusion, only the index stroke admission for each patient between 2019 and 2023 was included.

Patients were excluded if data on age, AF, or National Institutes of Health Stroke Scale (NIHSS) score were missing. Patients aged < 40 years were excluded due to the higher prevalence of uncommon stroke etiologies in this age group, which could confound the association between age and AF on stroke severity.

### Exposure and outcome variables

Age was determined from physician documentation in the stroke clinical pathway note at the time of admission. AF was defined as any documented AF recorded in physician notes within stroke clinical pathway notes or captured through ICD-10-AM diagnostic coding. AF subtype (paroxysmal, persistent, or permanent) and the specific diagnostic modality (e.g., electrocardiogram (ECG), telemetry) or whether AF was previously known or newly detected during the index admission were not distinguished in the dataset. All patients underwent a 12-lead electrocardiogram on presentation. Most patients received at least 6 h of continuous cardiac telemetry monitoring during the first day of admission. Additional inpatient rhythm monitoring, including 24-h Holter monitoring with the option of extended 48-h monitoring, was performed at the discretion of the managing physician.

Stroke severity was assessed using the NIHSS score. For each patient, the NIHSS score was determined based on the best available documentation using the following predefined order of priority: physician documentation of NIHSS on admission in the stroke clinical pathway admission notes, nursing documentation of “NIHSS on admission” in the stroke pathway flowsheet, nursing documentation of “NIHSS on admission” in the NIHSS monitoring flowsheet, then first NIHSS recorded in the NIHSS monitoring flowsheet. If the NIHSS score was not available in any of these sources, the field was left blank, and the patient was excluded from analysis.

Covariates appearing in the analysis included sex, race/ethnicity (Chinese, Malay, Indian, Other), and vascular risk factors or comorbidities [i.e., active malignancy, chronic kidney disease, diabetes mellitus, ischemic heart disease, previous myocardial infarction, hyperlipidemia, hypertension, and premorbid modified Rankin Scale (mRS)], as documented in the stroke pathway or coded using ICD-10-AM classifications.

### Bias

Selection bias was mitigated by including consecutive patients from our stroke registry and applying uniform inclusion and exclusion criteria. We used standardized information, including NIHSS, to assess stroke severity, ICD-10-AM diagnostic coding, and from predefined definitions of the stroke clinical pathway. We aimed to address confounding bias by adjusting for demographic and clinical factors in our interaction analysis.

### Ethical considerations

The analysis was based on de-identified data from an institutional stroke registry, and no identifiable patient information was accessible to investigators.

### Statistical analysis

Variables were summarized based on AF status with further stratification by age group. Given that NIHSS is an ordinal scale, stroke severity was summarized descriptively using median values with interquartile ranges, and non-parametric tests were used for unadjusted group comparisons. Regression-based modeling was subsequently employed to evaluate covariate-adjusted associations and interaction effects between age and atrial fibrillation. Data were presented as mean [standard deviation (SD)] or median [interquartile range (IQR)], whichever was appropriate, for continuous variables and as frequencies (percentages) for categorical variables. Differences between groups were tested using chi-squared test, analysis of variance (ANOVA) or Mann–Whitney *U* test, whichever was appropriate, for categorical and continuous variables, respectively. The independent variable age was categorized as a binary variable with categories < 65 and ≥65 years. Patients < 65 years old were defined as the *younger* age group, while the other group was defined as the *older* age group. We selected 65 years as the age cut-off because it represents a widely accepted clinical threshold in stroke and cardiovascular research that distinguishes younger from older adults in terms of vascular risk profile. Individuals aged more than 65 years generally have a higher burden of vascular comorbidities, making this a clinically relevant stratification for evaluating the interaction between age and AF on stroke severity (Joglar et al., 2024; Olesen et al., 2012). Stroke severity, as measured by the NIHSS, was the primary outcome and treated as a count variable. The NIHSS was modeled using a negative binomial distribution within PROC GLIMMIX with a logarithmic link function to account for overdispersion in the NIHSS scores (Pan et al., 2025). Although the NIHSS is an ordinal scale, its distribution in this study was highly right-skewed with variance exceeding the mean, making a negative binomial model a robust choice. The model included age, AF, and their interaction term, along with relevant covariates such as gender, race, and premorbid mRS. The result was reported as incidence rate ratios (IRRs) with 95% confidence interval (CI). An IRR greater than 1 signifies that the expected count of the NIHSS is higher by a factor equivalent to the IRR for a given covariate group compared to its reference group. Conversely, an IRR less than 1 indicates a lower expected count.

All tests were two-sided, and the statistical significance threshold was set at *p* < 0.05. All statistical analyses were performed using SAS version 9.4 (SAS Institute Inc., Cary, NC, USA).

## Results

### Study population

Of the 5,044 acute ischemic stroke patients (Table 1) included in the analysis, the mean age was 68.6 years (SD 12.0), and 62.8% were in the older age group. The majority were male (60.6%), and the ethnic distribution was Chinese (75.5%), Malay (9.4%), and Indian (8.9%). The median NIHSS score at admission was 2 (IQR 0–6). Among patients with available data on premorbid mRS (*n* = 4,620), 75.5% had a score of 0.

**Table 1:** Baseline characteristics of study population (*n* = 5,044).

**Characteristics**	**All patients *N* = 5,044**	**Patients with AF**			**Patients without AF**			***P*-value^b^**
		**Age**<**65 years** ***N*** = **120**	**Age** ≥**65 years** ***N*** = **755**	***P*** **-value** ^a^	**Age**<**65 years** ***N*** = **1,758**	**Age** ≥**65 years** ***N*** = **2,411**	***P*** **-value** ^a^	
Age (years), mean (SD)	68.6 (12.0)	58.2 (5.3)	78.5 (8.0)	0.10	56.0 (6.4)	75.3 (7.5)	< 0.001	< 0.001
NIHSS, median (IQR)	2.0 (0.0–6.0)	3.5 (0.0–11.0)	5.0 (1.0–15.0)	0.19	2.0 (0.0–4.0)	2.0 (0.0–5.0)	0.012	< 0.001
Male sex, *n* (%)	3,056 (60.6)	80 (66.7)	361 (47.8)	0.0001	1,230 (70.0)	1,385 (57.4)	< 0.001	< 0.001
Race, *n* (%)				0.0001			< 0.001	< 0.001
Chinese	3,810 (75.5)	79 (65.8)	618 (81.9)		1,161 (66.1)	1,951 (80.9)		
Indian	447 (8.9)	7 (5.8)	35 (4.6)		236 (13.4)	169 (7.0)		
Malay	473 (9.4)	25 (20.8)	63 (8.3)		212 (12.1)	173 (7.2)		
Others	314 (6.2)	079 (65.8)	618 (81.9)		1,161 (66.1)	1,951 (80.9)		
Premorbid mRS = 0, *n* (%)^*^	3,487 (75.5)	81 (75.0)	436 (62.0)	0.053	1,371 (84.5)	1,599 (73.1)	< 0.001	< 0.001
Active malignancy, *n* (%)	208 (4.1)	3 (2.5)	39 (5.2)	0.21	49 (2.8)	117 (4.9)	< 0.001	0.0028
Chronic kidney disease, *n* (%)	365 (7.2)	12 (10.0)	99 (13.1)	0.34	71 (4.0)	183 (7.6)	< 0.001	< 0.0001
Diabetes mellitus, *n* (%)	1,354 (26.8)	25 (20.8)	221 (29.3)	0.056	464 (26.4)	644 (26.7)	0.82	0.20
Ischemic heart disease, *n* (%)	728 (14.4)	31 (25.8)	181 (24.0)	0.66	186 (10.6)	330 (13.7)	0.003	< 0.0001
Previous MI, *n* (%)	284 (5.6)	13 (10.8)	68 (9.0)	0.52	79 (4.5)	124 (5.1)	0.34	< 0.0001
Hyperlipidemia, *n* (%)	3,073 (60.9)	72 (60.0)	485 (64.2)	0.37	1,009 (57.4)	1,507 (62.5)	0.001	0.002
Hypertension, *n* (%)	3,658 (72.5)	75 (62.5)	610 (80.8)	< 0.0001	1,102 (62.7)	1,871 (77.6)	< 0.0001	< 0.0001

^*^Out of 4,620 patients with available premorbid mRS data. AF: atrial fibrillation; NIHSS: National Institutes of Health Stroke Scale; SD: standard deviation; IQR: interquartile range. ^a^P-values were used to compare differences in two groups and are based on chi-squared test for categorical and independent t-test or Mann-Whitney U test, whichever was appropriate, for continuous data. ^b^P-values were used to compare the differences across the four groups and are based on chi-squared test for categorical and analysis of variance (ANOVA) or Kruskal–Wallis test, whichever appropriate, for continuous data.

AF was present in 875 (17.3%) patients. Gender, race, and all other comorbidities except diabetes were significantly different between the four groups stratified by age and AF status (young with AF, young without AF, old with AF, and old without AF).

**Figure 2:**
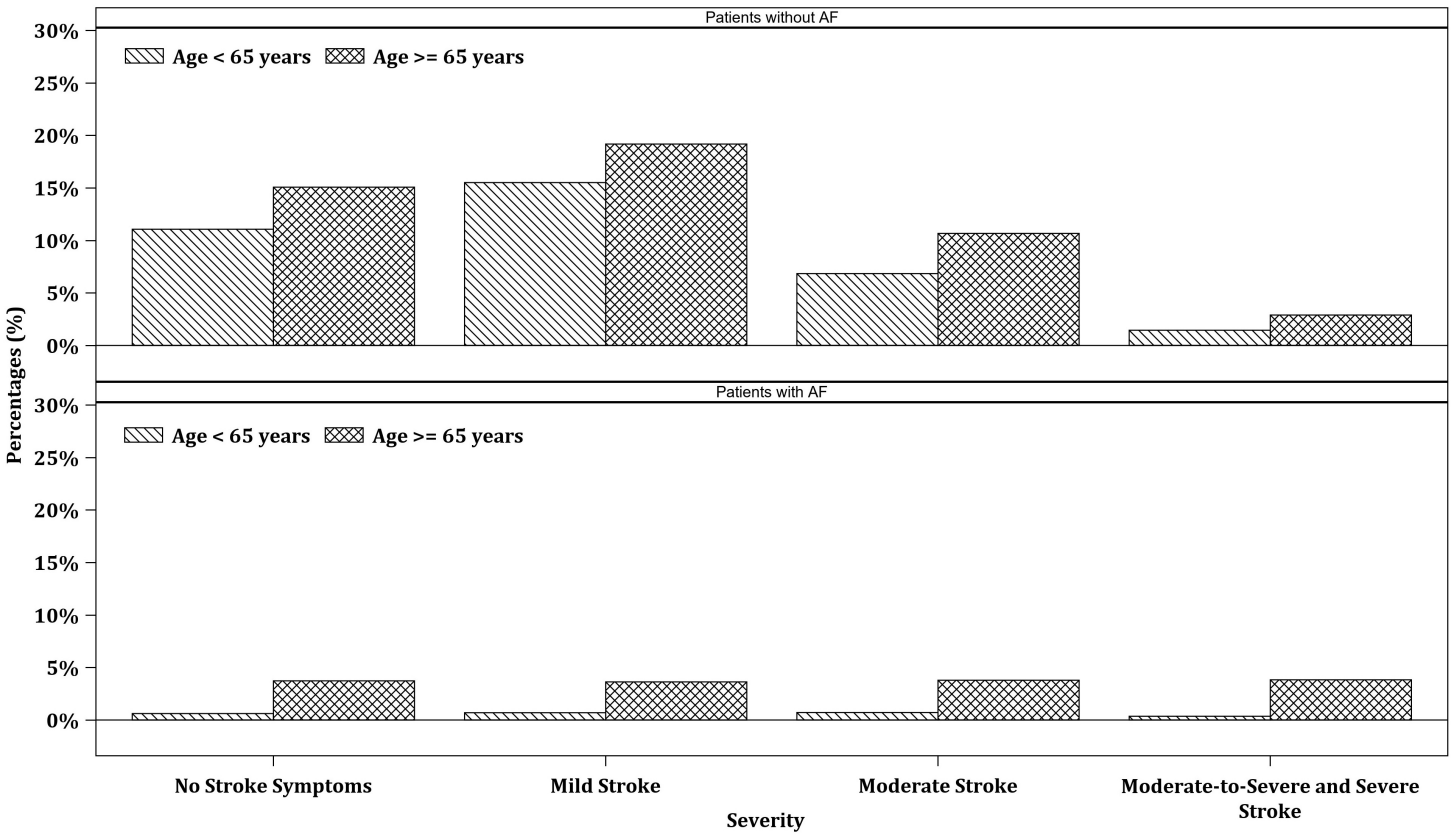
Distribution of stroke severity by NIHSS categories stratified by AF status and age group.

The distribution of stroke severity, based on NIHSS categories (no stroke symptoms: NIHSS = 0; mild stroke: NIHSS = 1–4; moderate stroke: NIHSS = 5–15; moderate to severe and severe stroke: NIHSS > 15), is presented in Figure 2. Among patients without AF, older patients (≥65 years) showed a higher proportion of mild, moderate, and moderate to severe/severe strokes compared to younger patients (< 65 years; *p* = 0.0017). In contrast, among patients with AF, stroke severity was more evenly distributed across age groups, with no statistically significant difference observed (*p* = 0.0814).

### Interaction between age and AF on stroke severity

Interaction between AF and age group was not significantly associated with NIHSS (*P* for interaction = 0.88; Table 2). Mean NIHSS scores were higher among older patients compared to younger patients, regardless of AF status. Among non-AF patients, older patients had a mean NIHSS score of 3.87 (0.11) compared to 3.18 (0.11) in younger patients (IRR: 1.13; 95% CI: 1.03–1.24; *p* = 0.0088). Among AF patients, older patients had a mean NIHSS of 8.19 (0.40) compared to 6.89 (0.85) in younger patients (IRR: 1.28; 95% CI: 0.97–1.69; *p* = 0.0767). Within both age groups, stroke severity was significantly higher in patients with AF compared to those without AF. In younger patients, the mean NIHSS was 6.89 (0.85) for those with AF vs. 3.18 (0.11) for those without AF (IRR: 1.88; 95% CI: 1.44–2.45; *p* < 0.0001). In older patients, the mean NIHSS score was 8.19 (0.40) for those with AF vs. 3.87 (0.11) for those without AF (IRR: 2.13; 95% CI: 1.89–2.39; *p* < 0.0001). After adjusting for age, gender, and premorbid mRS, there was no interaction between AF and age group (*P* for interaction = 0.40).

**Table 2:** Interaction of age and atrial fibrillation on National Institutes of Health stroke scale (NIHSS) score.

**(A) Comparing presence and absence of atrial fibrillation within different age groups**						
**Age group**	**Mean NIHSS (SE) (AF)**	**Mean NIHSS (SE)** **(No AF)**	**IRR (95% CI)**	***P*** **-value**	**IRR** ^*^ **(95% CI)**	*P* ^*^ **-value**
< 65 years	6.89 (0.85)	3.18 (0.11)	2.16 (1.68, 2.78)	< 0.0001	1.88 (1.44–2.45)	< 0.0001
≥65 years	8.19 (0.40)	3.87 (0.11)	2.12 (1.90, 2.37)	< 0.0001	2.13 (1.89–2.39)	< 0.0001
**(B) Comparing different age groups within presence and absence of atrial fibrillation**						
**AF status**	**Mean NIHSS (SE) (**<**65 years)**	**Mean NIHSS (SE)** **(**≥**65 years)**	**IRR (95% CI)**	***P*** **-value**	**IRR** ^×^ **(95% CI)**	*P* ^×^ **-value**
AF present	6.89 (0.85)	8.19 (0.40)	1.19 (0.92, 1.54)	0.1925	1.28 (0.97–1.69)	0.0767
AF absent	3.18 (0.11)	3.87 (0.11)	1.21 (1.11, 1.32)	< 0.0001	1.13 (1.03–1.24)	0.0088

SE, standard error; CI, confidence interval. Negative binomial regression models were employed for the count-based dependent variable NIHSS. ^*^Covariates race, gender, and premorbid mRS adjusted negative binomial regression model for the outcome NIHSS. Incidence rate ratios (IRRs) are provided; IRR > 1 indicates that the expected count of the NIHSS is higher for that group by a factor equal to the IRR, relative to the reference group. Conversely, IRR < 1 indicates a lower expected count relative to the reference group. Patients without AF or younger patients, i.e., patients aged < 65 years are treated as reference group. P (interaction between AF and age) = 0.88. P^×^ (interaction between AF and age) = 0.40.

## Discussion

In this study, we examined the interaction between age and AF on stroke severity, using NIHSS score as the outcome measure. The study yielded two key findings. First, AF was associated with significantly higher stroke severity in both younger and older patients. Notably, the magnitude of this effect was substantial in both age groups, with mean NIHSS scores nearly doubled in AF patients compared to non-AF patients. Second, there was no statistically significant interaction between age and AF status on NIHSS scores, indicating no evidence of effect modification by age within the limits of our data. These findings were not explained by differences in baseline characteristics, as adjustments were made for age, sex, and premorbid functional status.

Prior studies consistently demonstrated that AF was associated not only with an increased risk of ischemic stroke but also with greater stroke severity and higher mortality. Several pathophysiological mechanisms underlie the observed increase in stroke severity among patients with AF. AF-related strokes are cardioembolic, which are associated with larger volume infarction (Bogousslavsky et al., 1988; Heinsius et al., 1998; Tomita et al., 2000). Additionally, reductions in cerebral perfusion have been observed in patients with AF compared to those in sinus rhythm, potentially contributing to worse ischemic burden (Lavy et al., 1980). Age and NIHSS score were independently associated with poor outcome for ischemic stroke patients with AF in the early recovery stage (Li et al., 2013). Recent data further reinforce the strong association between AF and greater neurological deficit, significantly higher 30-day and 1-year mortality than non-AF patients at presentation driven by stroke severity (Vinding et al., 2022; Toyoda et al., 2025). Our findings also align with emerging evidence that AF-related strokes are clinically aggressive across diverse populations. Multicenter data from alteplase-treated AF patients show that higher admission NIHSS is a strong and independent predictor of all subtypes of hemorrhagic transformation and that sustained AF further contributes to poorer long-term outcomes (Ahmed et al., 2025; Zeinhom et al., 2025). Beyond the acute phase, AF patients continue to carry substantial long-term stroke risk on top of initial stroke severity. A large meta-analysis showed the incidence of recurrence after AF-related stroke was 3.75% per year despite anticoagulation, underscoring the substantial long-term vulnerability of this population (McCabe et al., 2025).

However, few studies have directly assessed whether the effect of AF on stroke severity differs by age. To our knowledge, this is among the first studies to directly evaluate the interactive effect of age and AF on initial stroke severity, using interaction modeling to assess age-modified differences in initial NIHSS scores. In current clinical practice, younger patients with AF may be underrecognized due to the lower absolute prevalence of stroke in this group. However, younger patients with AF face a disproportionately higher long-term risk of stroke compared to older patients with AF, particularly within the first year of diagnosis, and often bear a greater treatment burden (Hindricks et al., 2020; Karagianni et al., 2025; Cheng et al., 2025). This underrecognition is further compounded by limitations of current risk stratification tools used to guide anticoagulation decisions for primary stroke prevention. In particular, young patients without additional CHA_2_DS_2_-VASc risk factors are categorized as low thromboembolic risk, and current guidelines therefore do not recommend anticoagulation (January et al., 2019; Chao et al., 2012). These assessments focus on risk incidence rather than the severity of potential strokes. Our findings highlight that both younger and older patients with AF experience significantly more severe strokes compared to those without AF. As such, current risk stratification tools may insufficiently capture the true clinical burden, particularly in younger patients who, despite lower stroke incidence, remain at risk of disabling events. Thus, in counseling younger patients with AF, the severity of stroke and its consequences on long-term disability and complications, albeit lower incidence compared to older patients, should be discussed. The absence of a statistically significant interaction in our analysis suggests that the effect of AF on initial stroke severity does not appear to differ markedly by age group, although smaller or non-linear differences cannot be excluded.

This study has several limitations. First, the cross-sectional study design precluded causal inference between AF, age, and stroke severity. However, the use of a prospectively collected registry improved data quality, minimized recall bias, and ensured standardized variable definitions. Second, unmeasured confounders, such as AF subtype and duration, anticoagulation status, time to presentation, and the presence of previous stroke or TIA, could have influenced the observed associations. Data on stroke subtype and infarct territory were not available, limiting our ability to determine whether the severity differences were mediated by specific stroke mechanisms. Third, AF was defined as documented during admission, and patients with paroxysmal or newly detected AF after discharge might have been misclassified. Fourth, the statistically non-significant interaction between age and AF should not be interpreted as evidence of equivalent effects across age groups. Rather, it indicates that we did not detect effect modification within the limits of our sample size and statistical power. Residual confounding or limited power to detect interaction effects cannot be excluded, and the findings should therefore be interpreted cautiously. Finally, although NIHSS is a validated measure of stroke severity, it may underrepresent deficits related to posterior circulation or cognition and may not fully capture clinical burden across all stroke subtypes.

Our findings are largely applicable to patients with acute ischemic stroke treated in tertiary hospitals with comparable demographic and clinical characteristics. However, there may be considerations with extrapolation to other populations with different characteristics including ethnicity, healthcare access, and anticoagulation practices.

## Conclusion

In summary, this study demonstrated that the association between AF and increased stroke severity was evident in both younger and older adults. There was no statistically significant interaction between age and AF status on NIHSS scores, indicating no evidence of effect modification by age within the limits of our data.

## Data Availability

The original contributions presented in the study are included in the article/Supplementary material, further inquiries can be directed to the corresponding author.
