# Supplementary material for: Interaction between age and atrial fibrillation on ischemic stroke severity: a cross-sectional analysis
Source: Front Stroke. 2026 Feb 2;5:1706746. doi: 10.3389/fstro.2026.1706746 (PMC12907190; doi:10.3389/fstro.2026.1706746)
Supplement: Supplementary file 1 [file Table_1.pdf]

Supplementary Table 1. Comparison of Baseline Characteristics Between Included Patients and Those Excluded Due to Missing NIHSS.

Variable	--- Groups --- Included N = 5044	Not included N = 785	Total N = 5829	P value
Year, n(%)				< 0.0001
N#	5044	785	5829	
2019	1033 (20.5)	6 (0.8)	1039 (17.8)	
2020	936 (18.6)	43 (5.5)	979 (16.8)	
2021	976 (19.3)	268 (34.1)	1244 (21.3)	
2022	1058 (21.0)	217 (27.6)	1275 (21.9)	
2023	1041 (20.6)	251 (32.0)	1292 (22.2)	
Age				< 0.0001
N#	5044	785	5829	
Mean (SD)	68.6 (12.0)	73.0 (12.0)	69.2 (12.1)	
Median (IQR)	69.0 (17.0)	74.0 (17.0)	69.0 (17.0)	
Min, Max	40, 103	40, 102	40, 103	
Missing, n(%)	0	0	0	
AF, n(%)				0.5872
N#	5044	785	5829	
0	4169 (82.7)	655 (83.4)	4824 (82.8)	
1	875 (17.3)	130 (16.6)	1005 (17.2)	
Gender, n(%)				0.0131
N#	5044	785	5829	
Female	1988 (39.4)	346 (44.1)	2334 (40.0)	
Male	3056 (60.6)	439 (55.9)	3495 (60.0)	

Variable	--- Groups --- Included N = 5044	Not included N = 785	Total N = 5829	P value
Race, n(%)				0.3087
N#	5044	785	5829	
Bangladeshi	9 (0.2)	1 (0.1)	10 (0.2)	
Burmese	4 (0.1)	3 (0.4)	7 (0.1)	
Butonese	1 (0.0)	0	1 (0.0)	
Caucasian	22 (0.4)	1 (0.1)	23 (0.4)	
Chinese	3809 (75.5)	605 (77.1)	4414 (75.7)	
Eurasian	19 (0.4)	2 (0.3)	21 (0.4)	
Filipino	40 (0.8)	4 (0.5)	44 (0.8)	
Indian	447 (8.9)	75 (9.6)	522 (9.0)	
Indonesian	43 (0.9)	2 (0.3)	45 (0.8)	
Japanese	8 (0.2)	1 (0.1)	9 (0.2)	
Korean	2 (0.0)	0	2 (0.0)	
Malay	473 (9.4)	67 (8.5)	540 (9.3)	
NA	1 (0.0)	0	1 (0.0)	
Nepalese	0	1 (0.1)	1 (0.0)	
Other Races	128 (2.5)	17 (2.2)	145 (2.5)	
Pakistani	6 (0.1)	1 (0.1)	7 (0.1)	
Sikh	22 (0.4)	3 (0.4)	25 (0.4)	
Sri Lankan	4 (0.1)	1 (0.1)	5 (0.1)	
Thai	2 (0.0)	1 (0.1)	3 (0.1)	
Vietnamese	4 (0.1)	0	4 (0.1)	
Active Maglinancy, n(%)				0.0576
N#	5044	785	5829	
No	4836 (95.9)	741 (94.4)	5577 (95.7)	
Yes	208 (4.1)	44 (5.6)	252 (4.3)	
Chronic Kidney Disease, n(%)				<.0001
N#	5044	785	5829	
No	4679 (92.8)	770 (98.1)	5449 (93.5)	
Yes	365 (7.2)	15 (1.9)	380 (6.5)	
Diabetes, n(%)				<.0001
N#	5044	785	5829	
No	3690 (73.2)	726 (92.5)	4416 (75.8)	
Yes	1354 (26.8)	59 (7.5)	1413 (24.2)	
Ischemic Heart Disease, n(%)				<.0001
N#	5044	785	5829	
No	4316 (85.6)	750 (95.5)	5066 (86.9)	
Yes	728 (14.4)	35 (4.5)	763 (13.1)	

Variable	--- Groups --- Included N = 5044	Not included N = 785	Total N = 5829	P value
Previous MI, n(%)				0.0737
N#	5044	785	5829	
No	4760 (94.4)	753 (95.9)	5513 (94.6)	
Yes	284 (5.6)	32 (4.1)	316 (5.4)	
Hyperlipidemia, n(%)				<.0001
N#	5044	785	5829	
No	1971 (39.1)	380 (48.4)	2351 (40.3)	
Yes	3073 (60.9)	405 (51.6)	3478 (59.7)	
Hypertension, n(%)				<.0001
N#	5044	785	5829	
No	1386 (27.5)	269 (34.3)	1655 (28.4)	
Yes	3658 (72.5)	516 (65.7)	4174 (71.6)	
MRS Premorbid, n(%)				<.0001
N#	4620	109	4729	
0	3487 (75.5)	65 (59.6)	3552 (75.1)	
1	636 (13.8)	17 (15.6)	653 (13.8)	
2	216 (4.7)	4 (3.7)	220 (4.7)	
3	122 (2.6)	8 (7.3)	130 (2.7)	
4	137 (3.0)	13 (11.9)	150 (3.2)	
5	22 (0.5)	2 (1.8)	24 (0.5)	
